# Supplementary material for: Maternal pre-pregnancy body mass index and related factors: A cross-sectional analysis from the Japan Environment and Children’s Study
Source: PLoS One. 2024 Jun 4;19(6):e0304844. doi: 10.1371/journal.pone.0304844 (PMC11149848; doi:10.1371/journal.pone.0304844)
Supplement: S1 Table — (DOCX) [file pone.0304844.s001.docx]

Supplemental Table 1. Relative ratios of age, parity, smoking, and socioeconomic factors by BMI class (complete dataset, N=78,682)

|  | Severe-moderate underweight | | | Mild underweight | | | Normal | Overweight | | | Obesity | | |
| --- | --- | --- | --- | --- | --- | --- | --- | --- | --- | --- | --- | --- | --- |
|  | aRR | 95%CI | P | aRR | 95%CI | P | Reference | aRR | 95%CI | P | aRR | 95%CI | P |
| Age (years) |  |  |  |  |  |  |  |  |  |  |  |  |  |
| <19 | 1.85 | (1.27-2.70) | 0.001 | 1.13 | (0.88-1.44) | 0.334 | - | 0.58 | (0.41-0.81) | 0.002 | 0.23 | (0.10-0.49) | <0.001 |
| 20-24 | 1.34 | (1.15-1.57) | <0.001 | 1.34 | (1.24-1.45) | <0.001 | - | 0.74 | (0.66-0.82) | <0.001 | 0.54 | (0.44-0.65) | <0.001 |
| 25-29 | 1.23 | (1.11-1.38) | <0.001 | 1.14 | (1.08-1.20) | <0.001 | - | 0.84 | (0.79-0.90) | <0.001 | 0.76 | (0.68-0.86) | <0.001 |
| 30-34 | 1.00 |  |  | 1.00 |  |  |  | 1.00 |  |  | 1.00 |  |  |
| 35-39 | 0.68 | (0.59-0.78) | <0.001 | 0.84 | (0.79-0.89) | <0.001 | - | 1.15 | (1.07-1.23) | <0.001 | 1.19 | (1.06-1.34) | 0.004 |
| >40 | 0.53 | (0.38-0.74) | <0.001 | 0.74 | (0.65-0.85) | <0.001 | - | 1.36 | (1.20-1.55) | <0.001 | 1.17 | (0.93-1.47) | 0.190 |
| Parity |  |  |  |  |  |  |  |  |  |  |  |  |  |
| 0 | 1.00 |  |  | 1.00 |  |  |  | 1.00 |  |  | 1.00 |  |  |
| 1 | 0.85 | (0.77-0.95) | 0.004 | 0.99 | (0.94-1.04) | 0.569 | - | 1.13 | (1.06-1.20) | <0.001 | 1.04 | (0.93-1.16) | 0.518 |
| >2 | 0.81 | (0.70-0.92) | 0.002 | 0.91 | (0.85-0.97) | 0.002 | - | 1.15 | (1.07-1.23) | <0.001 | 1.09 | (0.96-1.23) | 0.182 |
| Smoking |  |  |  |  |  |  |  |  |  |  |  |  |  |
| Never-smoking | 1.00 |  |  | 1.00 |  |  |  | 1.00 |  |  | 1.00 |  |  |
| Quitting smoking before pregnancy | 0.84 | (0.75-0.95) | 0.004 | 0.88 | (0.83-0.93) | <0.001 | - | 1.03 | (0.97-1.10) | 0.327 | 1.14 | (1.02-1.27) | 0.023 |
| Quitting smoking early pregnancy/still smoking | 1.29 | (1.15-1.45) | <0.001 | 1.05 | (0.99-1.11) | 0.135 | - | 1.20 | (1.12-1.29) | <0.001 | 1.34 | (1.19-1.51) | <0.001 |
| Marital status |  |  |  |  |  |  |  |  |  |  |  |  |  |
| Married | 1.00 |  |  | 1.00 |  |  |  | 1.00 |  |  | 1.00 |  |  |
| Unmarried | 1.08 | (0.86-1.37) | 0.495 | 1.16 | (1.03-1.31) | 0.016 | - | 0.99 | (0.84-1.17) | 0.938 | 0.86 | (0.64-1.15) | 0.303 |
| Divorced or bereavement | 1.03 | (0.64-1.68) | 0.892 | 1.45 | (1.16-1.81) | 0.001 | - | 0.81 | (0.62-1.07) | 0.136 | 0.60 | (0.38-0.95) | 0.030 |
| Occupational status |  |  |  |  |  |  |  |  |  |  |  |  |  |
| Employed | 1.00 |  |  | 1.00 |  |  |  | 1.00 |  |  | 1.00 |  |  |
| Housewife or unemployed | 1.25 | (1.13-1.38) | <0.001 | 1.11 | (1.06-1.17) | <0.001 | - | 1.02 | (0.96-1.07) | 0.604 | 1.15 | (1.04-1.26) | 0.006 |
| Student | 0.70 | (0.37-1.33) | 0.278 | 0.98 | (0.73-1.33) | 0.910 | - | 0.69 | (0.42-1.15) | 0.158 | 0.75 | (0.27-2.07) | 0.582 |
| Household income (million yen) |  |  |  |  |  |  |  |  |  |  |  |  |  |
| <199 | 0.99 | (0.75-1.31) | 0.944 | 0.91 | (0.80-1.05) | 0.203 | - | 1.67 | (1.4-2.00) | <0.001 | 3.14 | (2.21-4.46) | <0.001 |
| 200-399 | 0.94 | (0.75-1.18) | 0.608 | 0.89 | (0.80-0.99) | 0.039 | - | 1.40 | (1.21-1.63) | <0.001 | 2.06 | (1.49-2.84) | <0.001 |
| 400-599 | 0.80 | (0.63-1.00) | 0.051 | 0.86 | (0.77-0.95) | 0.004 | - | 1.22 | (1.05-1.41) | 0.010 | 1.66 | (1.21-2.29) | 0.002 |
| 600-799 | 0.78 | (0.62-1.00) | 0.052 | 0.86 | (0.77-0.96) | 0.009 | - | 1.08 | (0.92-1.26) | 0.340 | 1.36 | (0.97-1.91) | 0.070 |
| 800-899 | 0.79 | (0.59-1.05) | 0.102 | 0.96 | (0.84-1.09) | 0.502 | - | 0.99 | (0.83-1.19) | 0.952 | 1.23 | (0.84-1.80) | 0.292 |
| >1,000 | 1.00 |  |  | 1.00 |  |  |  | 1.00 |  |  | 1.00 |  |  |
| Educational attainment |  |  |  |  |  |  |  |  |  |  |  |  |  |
| ECD1 | 1.29 | (1.03-1.63) | 0.027 | 0.96 | (0.85-1.08) | 0.488 | - | 2.19 | (1.91-2.52) | <0.001 | 4.01 | (3.19-5.04) | <0.001 |
| ECD2 | 1.12 | (0.97-1.28) | 0.116 | 0.90 | (0.84-0.95) | 0.001 | - | 1.80 | (1.66-1.96) | <0.001 | 2.71 | (2.31-3.19) | <0.001 |
| ECD3 | 1.02 | (0.90-1.15) | 0.795 | 0.93 | (0.88-0.98) | 0.009 | - | 1.43 | (1.32-1.54) | <0.001 | 1.72 | (1.47-2.01) | <0.001 |
| ECD4 | 1.00 |  |  | 1.00 |  |  |  | 1.00 |  |  | 1.00 |  |  |

BMI: Body mass index

aRR: adjusted relative ratio

CI: confidence interval

ECD1: Junior high school; ECD2: High school; ECD3: Technical junior college, technical/vocational college, or associate degree; ECD4: bachelor’s degree or postgraduate degree.
